# DirtyGenes: testing for significant changes in gene or bacterial population compositions from a small number of samples

**DOI:** 10.1038/s41598-019-38873-4

**Published:** 2019-02-20

**Authors:** Laurence M. Shaw, Adam Blanchard, Qinglin Chen, Xinli An, Peers Davies, Sabine Tötemeyer, Yong-Guan Zhu, Dov J. Stekel

**Affiliations:** 1 School of Biosciences, University of Nottingham, Loughborough, LE12 5RD UK; 2 School of Science and Technology, Nottingham Trent University, Nottingham, NG11 8NS UK; 3 School of Veterinary Medicine and Science, University of Nottingham, Loughborough, LE12 5RD UK; 4 School of Animal, Rural and Environmental Sciences, Nottingham Trent University, Brackenhurst, NG25 0FQ UK; 5 Chinese Academy of Sciences, Institute of Urban Environment, Xiamen, 361021 China; 6 Institute of Infection and Global Health, University of Liverpool, Liverpool, L69 7BE UK

## Abstract

High throughput genomics technologies are applied widely to microbiomes in humans, animals, soil and water, to detect changes in bacterial communities or the genes they carry, between different environments or treatments. We describe a method to test the statistical significance of differences in bacterial population or gene composition, applicable to metagenomic or quantitative polymerase chain reaction data. Our method goes beyond previous published work in being universally most powerful, thus better able to detect statistically significant differences, and through being more reliable for smaller sample sizes. It can also be used for experimental design, to estimate how many samples to use in future experiments, again with the advantage of being universally most powerful. We present three example analyses in the area of antimicrobial resistance. The first is to published data on bacterial communities and antimicrobial resistance genes (ARGs) in the environment; we show that there are significant changes in both ARG and community composition. The second is to new data on seasonality in bacterial communities and ARGs in hooves from four sheep. While the observed differences are not significant, we show that a minimum group size of eight sheep would provide sufficient power to observe significance of similar changes in further experiments. The third is to published data on bacterial communities surrounding rice crops. This is a much larger data set and is used to verify the new method. Our method has broad uses for statistical testing and experimental design in research on changing microbiomes, including studies on antimicrobial resistance.

## Introduction

Bacteria live in complex communities, whether in water, in soil, or on larger organisms, as the microbiota of organs such as the gut or the skin. New high throughput technologies, including high throughput sequencing^1^, or qPCR arrays^2^, allow for the characterisation of microbial communities^3^, or the genes that they carry^4^. This has broad application across biomedical and environmental science, and in particular, allows for detection of changes in communities or the genes that they carry in the face of biological^5^, chemical^6^ or environmental^7,8^ factors. Of particular relevance are studies on antimicrobial resistance^4,6,7,9–11^, as well as studies in other areas, such as changes in gut flora in different human communities^12^ or through the ingestion of probiotics^13^.

For robust reporting of research, it is important to use statistics correctly to ascertain whether there is evidence that the observed changes in the taxonomic or genetic composition of a community reflect the factors under study, as opposed to merely reflecting random variation between the samples. By *composition*, we refer to the proportions of individual taxanomic or gene classes within the population; this can be contrasted with *abundance*, which refers to the number of individuals, either overall, or of specific taxanomic or gene classes, or *overall structure*, which takes into account both abundance and composition. However, much previous work, especially in antimicrobial resistance, has largely focussed on visual methods for analysing data, using tables and graphical representations to compare overall population compositions or structures^4,6,7,10,14^, without providing statistical support to evidence change. Methods such as taking diversity indices or principal coordinate analysis^11^ have allowed for a more in depth analysis of population structures than using pie charts/compositional bar charts. However, these techniques are also visual, and do not have a clear blueprint to determine whether observed differences in structure are significant.

There are methods available to answer similar questions. For example, methods developed for cDNA library^15^ or RNA-seq analysis^16–18^, though not commonly used in this context, could be applied to metagenomics data on a taxon-by-taxon or gene-class-by-gene-class basis to identify individual taxa or gene classes that are significantly different. While such analyses could be of value, they do not answer the question about whether the overall community has changed. Moreover, such analyses would be subject to considerable numbers of false positives because of the number of tests applied, and would be difficult to correct for multiple testing because of the high level of correlation between different taxanomic or gene classes.

To date, only one method has been developed to answer the question posed in this paper^19^. La Rosa *et al*. model entire populations of metagenomic data according to a *Dirichlet*-*multinomial* distribution; surprisingly, however, their method uses Wald’s test to determine p-values, which is less reliable than likelihood ratio tests or scores tests, especially for smaller sample sizes (*n* < 10), which are common in very many studies^20^. Moreover, it is long established that likelihood ratio tests are more generally appropriate than Wald’s test, because they have the property of being universally most powerful: for a fixed false positive error rate, likelihood ratio tests provide the smallest rate of false negative errors.

A related problem is experimental design. Power analyses are routinely used to determine how many individuals to recruit into experimental studies. However, power analyses require a given statistical method for analysis of the resulting data. The PERMANOVA method of^21^ uses a non-parametric approach for estimating sample sizes for 16S microbiome studies, while Matiello *et al*.^22^ use the method of La Rosa *et al*.^19^ to estimate sample sizes for case control studies. While extremely useful, these methods have either limited scope, or are underpowered, so will tend to overestimate the number of samples needed. These matters are addressed in the methods we develop.

In this work, we develop new statistical tests to determine whether the taxanomic or gene composition of microbial community samples are statistically different between two or more sets of treatments or conditions that are reliable for smaller sample sizes. By considering the composition of ARG or bacterial population data in terms of a *Dirichlet distrubution*^23^, we are able to perform a likelihood ratio test in order to obtain a p-value to determine the level of evidence that population compositions vary significantly across multiple environments. The likelihood ratio test has the property of being universally most powerful. This means that it will give more reliable p-values than methods based on Wald’s test^19^, especially for experiments with smaller number of samples; it will also give more reliable estimates for sample sizes in power analyses. Moreover, we allow for empirical p-value estimation using a randomization procedure, again improving the reliability of the p-values, especially in experiments with small numbers of replicates.

There are three reasons for using a Dirichlet distribution over a Dirichlet-multinomial distribution. First, we are developing a test that is applicable to all types of data, including those without counts, such as qPCR arrays, as well as sequence data with counts. The second is that likelihood ratio test is much more computationally intensive than the Wald’s test for a Dirichlet-multinomial distribution, particularly if the count data is very high (as is often the case when considering gene/taxa counts); the Dirichlet distribution negates this issue by making all data compositional. Finally, with the Dirichlet distrbution, each sample is given an equal weighting, as opposed to those with larger counts most influencing the test; this means that artefacts in sequencing, such as variability in DNA preparation, will not bias the results.

The main drawback of using a Dirichlet over a Dirichlet-multinomial distribution is that the Dirichlet-multinomial includes an additional parameter that can describe intra-class correlation; the Dirichlet assumes that pairs of classes are uncorrelated. However, we include in our methods a goodness-of-fit test to determine whether the Dirichlet distribution provides a reasonable model for a given dataset, without which the significance test outlined would not be valid. The example data sets pass this goodness of fit test, so the Dirichlet distribution is appropriate.

As examples, we apply the method to two small data sets: soil microbiome data following manure amendment^11^, that uses qPCR arrays and 16S metagenomics; and previously unpublished data from a pilot study on seasonal changes in the microbiota of ovine hooves, that uses shotgun metagenomics. The interdigital skin environment of cloven hoofed animals has a dynamic bacterial community, composed mainly of skin and faecal colonising bacteria, but also soil microbes^24,25^. We also use a larger data set to verify our methods, looking at the bacterial composition of the rhizosphere of rice crops across two site^8^. Moreover, we also show how this test can be used for experimental design, in order to identify numbers of individuals to use in an experiment. These exemplify the broad applicability of this approach.

## Results

We first briefly describe the test statistic used to compare populations across multiple environments, before conducting two example analyses. In both examples, the sample sizes for each environment is small, and thus the combination of likelihood ratio test with empirical p-value determination is more reliable than the use of Wald’s test. A more detailed derivation of the test statistic is given in the Methods section which can be applied to any multi-type population, including bacterial populations.

Consider the profiles of samples taken from a set of different treatments or environments with replication. These profiles could be proportions of taxanomic groups (at any level) or proportion of gene groups, for example classes of antibiotic resistance genes. We test the null hypothesis, which states that any differences in composition between treatments/environments are a result of pure chance, against the alternative hypothesis that the population composition is affected by the treatment/environment.

We classify the composition of the taxanomic or genetic groups by a *Dirichlet distribution*. Under the null hypothesis, the composition of every sample is drawn from the same Dirichlet distribution. Under the alternative hypothesis, the parameterisation of the Dirichlet distribution governing the composition of the groups changes according to the treatment or environment.

The test statistic takes the form

$$D=-\,2\,\mathrm{log}\,(\frac{{L}_{0}}{{L}_{1}}).$$

where *L*_0_ and *L*_1_ are the maximum values of the likelihood function for the Dirichlet distribution parameterisation under the null and alternative hypotheses respectively. In the limit of a large number of samples, under the null hypothesis, *D* would follow a ${\chi }_{(m-1)K}^{2}$ distribution where *m* is the number of environments in the study and *K* is the number of ARG classes. However, most experiments do not have sufficient replication for this approximation to be valid. Therefore we use a randomization procedure to determine p-values. Further details on the derivation and calculation of the test statistic, and of the randomization procedure, are in the Methods section.

### Example Analysis: Sewage Sludge, China

Our first data set considers the effect of the application of sewage sludge to soil^11^. ARG and bacteria populations were profiled under eight different soil treatments with three samples being taken from each soil type (giving a total of 24 samples). Following the labelling of Chen *et al*. 2016, the soil types are referred to as follows: *CK* is a control soil containing no manure or urea (a chemical fertiliser); *0*.*5N* and *1N* soils contained urea with double the application rate in the 1N soil; *CM* contained chicken manure; while *0*.*5SS*, *1SS*, *2SS* and *4SS* contained sewage sludge with the application rate doubling with the coefficient. All the manure soils also contained the same application of urea as 0.5N.

**Figure 1:**
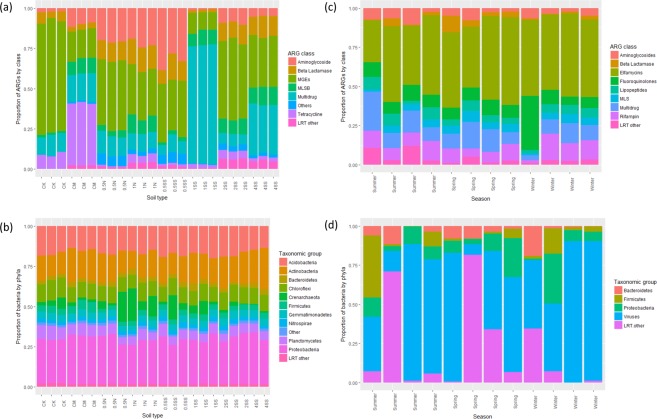
Compositional data of (**a**) ARGs by class and (**b**) bacterial taxa by phyla for each of the soil samples; similarly (**c**) ARGs by class for each of the sheep and (**d**) bacterial taxa by phyla. By visual inspection, there appear to be differences between ARGs between the different soil treatments but it us not clear whether the bacterial communities are different. In (**c** and **d**) there is greater variation between individuals and it is difficult to see whether there are differences between the seasons.

Figure 1(a) displays the compositional data of the ARGs by the class of drug to which they are resistant and Fig. 1(b) shows bacteria by phyla for each of the samples from these data. Note that results from one of the 4SS samples was missing from the bacterial data. In each case, only ARG classes/bacterial phyla that were ever-present across all samples and accounted for at least 1% of the population in at least one sample were included. Classes/phyla not meeting these criteria were aggregated into a group called *LRT other*. The reasons for this are outlined in the Methods section.

Visual inspection of Fig. 1(a) suggests that the composition of ARGs by class shows little variation within the same soil types but does seem to change with the environment. However, it is not clear from Fig. 1(b) whether the soil environment has an affect on the composition of bacterial populations.

Applying our likelihood ratio test using the *χ*^2^ method gives p-values of 1.03 × 10^−125^ for the ARG data and 1.32 × 10^−19^ for the bacterial data. These results were validated using the randomization approach (5000 random samples) which found p-values of <0.0002 and 0.0266 for the ARG and bacterial data respectively.

This provides strong evidence to suggest that the composition of both ARG and bacteria populations are affected by soil amendment; the evidence is particularly strong for the ARG data. Goodness-of-fit testing supports the notion that the Dirichlet distribution provides an adequate model for both sets of compositional data, with p-values of 0.55 for the ARG data 0.479 for the bacteria data, using 10000 simulations. Note that it is these non-significant p-values that indicate that the fit is good. Details on how goodness-of-fit testing is performed for these data can be found in the Methods section.

### Sheep Hooves, UK

The second dataset looks at how sheep hooves provide a reservoir for the spread of AMR. Four different sheep were used and the ARG and bacterial populations on their hooves were profiled in three different seasons (Winter, Spring and Summer), giving 12 samples overall. Further details on how the data were collected are given in the Methods section. The small sample size per environment of *n* = 4 provides strong grounds for using the likelihood ratio test over a Wald’s test.

The compositional data of both the ARG and bacterial populations for each sample is shown in Fig. 1(c,d). Again, all classes/phyla that were not ever-present and did not account for at least 1% of at least one sample were amalgamated into the *LRT other* category.

For these data, visual inspection suggests that changing seasons does not explain much of the variation in either the ARG or bacterial population data. The Dirichlet likelihood ratio test using the chi-squared method gave p-values of 0.057 for the ARG data and 0.597 for the bacteria data. Randomization testing gave p-values of 0.586 and 0.898 respectively. In both cases the null hypothesis that season does not have a significant effect on population composition could not be rejected at the 5% level. However the disparity in p-values, especially for the ARG data suggests that we may not have enough samples to reliably use the *χ*^2^ method and that randomization method provides more appropriate p-values to quote in this case. Choosing between the randomization and *χ*^2^ approaches is discussed further in the Methods section. Goodness-of-fit testing gave p-values of 0.484 and 0.511 for the ARG and bacterial data respectively, again suggesting that our proposed Dirichlet model provides an adequate fit to these data.

### Rhizosphere of Rice Crops, USA

Finally, we consider the bacterial composition, by phyla, of the rhizosphere of rice crops across two different sites in the USA. The data uses part of the publicly available *S2 Data* file from the paper of Edwards *et al*.^8^. Within the paper, the authors show that root associated bacteria are determined by both the distance from the root and the growing region using the distribution free PCoA and PERMANOVA methods.

Since our method only considers one environmental factor at a time, we restrict the data to samples from a single growing region, the rhizosphere, and test whether site has an effect. This leaves us with 226 samples from Arbuckle, CA and 128 samples from Jonesboro, AK. Again we only considered phyla that were ever-present and accounted for at least 1% of the population in at least one sample.

The likelihood ratio test with the *χ*^2^ method returns a p-value of 2.81 × 10^−286^ providing strong evidence to agree with the original conclusion that site does have a clear effect on bacterial composition. Importantly, goodness-of-fit testing gave a p-value of 0.286 from 10000 simulations, suggesting that the Dirichlet distribution provides a reasonable fit to the data and showing that it is not necessary to apply less powerful, non-parametric methods such as PERMANOVA to these data. The randomization approach was not used on these data because of the large sample sizes, as outlined in the Methods section.

### Power Analysis

The test method can also be used for experimental design, through its use in power analysis to estimate group sizes in future experiments. This is achieved by assuming the alternative hypothesis that population composition is affected by the treatment or environment is true, and using the currently observed data as the *best guess* for the distribution of the population in each environment. Full details are given in the methods section.

**Figure 2:**
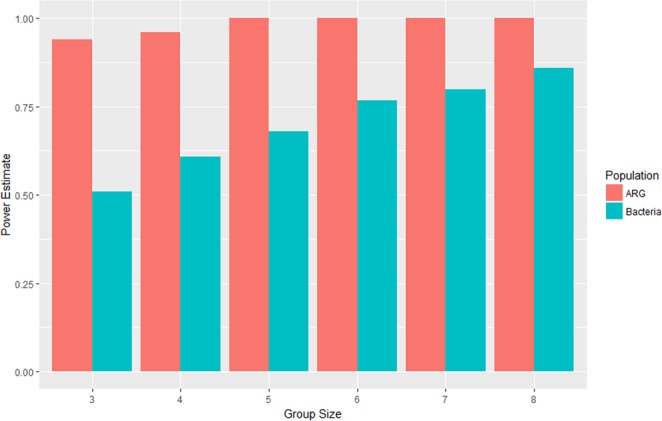
Power estimates of the Dirichlet LRT test for repeated experiments of the sheep hooves experiment with different numbers of sheep.

In the sheep hoof pilot study, we could not find significant evidence to support the hypothesis that ARG or bacterial population structures are affected by season; however, the pilot study from which the data derive only used a group size of four sheep for each season. Figure 2 shows estimates the power of future tests for groups containing up to 8 sheep for both the ARG and bacteria data, under the assumption that the observed differences in population composition are representative of genuine differences between the three seasons. The power estimates were generated using 500 simulations for each group size.

We observe that if there are differences between the ARG populations then future experiments should be very powerful at the 5% level regardless of the number of sheep used. (Recall the p-value close to 0.05 for the original data under the *χ*^2^ test). Creating an experiment with sufficient power to observe differences in bacterial populations between seasons requires more sheep: an experiment with a group of eight sheep should give us more than an 80% chance of finding significant evidence to support such a claim.

## Discussion

We have described and demonstrated a method for comparing gene or taxonomic composition of populations across a range of treatments or environments by modelling compositional population data in terms of a Dirichlet distribution and using a likelihood ratio test. Our method goes beyond existing methods^19^ through being more powerful and more generally applicable to a wider range of data types, including qPCR arrays. The method can also be used for estimating sample sizes for further experiments.

Our primary motivation and application has been to antimicrobial resistance, although the method is valid for any multi-type microbial community, as exemplified by the rhizosphere analysis. We have built on previous contributions to this field by describing differences in entire populations using a single test statistic, rather than relying on analysing one ARG/bacterial taxa at a time between different environments. This allows us to draw quick inferences as to whether changing environments are having an effect on the composition of our data (by simply reading off and interpreting a p-value) and removes the need to consider problems associated with making multiple comparisons^15^.

An important limitation of the test is to only include population types or classes that are present across all samples in the data, and that meet the criterion that the type accounts for at least 1% of the population in at least one of the samples. To counter this, a small background correction can be added to all classes^23^, so that the condition is met. Alternatively, all types not meeting both criterion need to be aggregated into an “other” class. One of these approaches is essential for the method to give reliable estimates for the parameters of the Dirichlet distributions. However, in most cases, the interest is in the differences between the more abundant classes within the data, and so this limitation is unlikely to have a major impact on interpretation of the data. The issues behind the limitation are explored in greater detail in the Further Considerations part of the Methods section.

The method outlined in this paper has the advantage being able to look at entire multitype populations (both in AMR and other fields) but does not use information on abundances, which may also provide an insight into differences between environments. Despite using a Dirichlet-multinomial distribution, which requires count data, La Rosa *et al*.^19^ do not look at abundances as a measure of differences between environments; they are simply used to estimate the parameters governing composition and overdispersion. A possible extension to both their and our method would be to model the overall structure of the observed populations using a Dirichlet-multinomial distribution associated with a probability vector *π* and an overdispersion parameter *θ* as before. The distribution would also need a number of trials, *N*, representing the overall size of the population, which would also be a random variable based on the distribution of the overall population sizes.

One would need to decide upon an appropriate distribution for *N* and also consider the issue that populations with larger *N* would contribute more to the likelihood function, meaning that each observation would not make an equal contribution to the likelihood ratio test statistic. However, such a method would use all of the information available from a multi-type population and, as already stated for Dirichlet-multinomial tests, would remove the problem needing ever-present types since zeros are supported within multinomial distributions.

For potential users of the test, we have produced R code which we have placed on GitHub (https://github.com/LMShaw/DirtyGenes), together with details of how to use the functions. Moreover, we foresee considerable potential for inclusion of this method into pipelines for analysis of antimicrobial resistance data^26,27^.

## Methods

This section contains a significant amount of mathematical notation. Table 1 contains a list of the key notation used for ease of reference.

**Table 1:** Key notation used in the Methods section.

Notation	Explanation
*K*	Number of classes in the multitype population being tested, labelled *k* = 1, 2, …, *K*.
*m*	Number of environments, labelled *i* = 1, 2, …, *m*
*n*_*i*_	Number of observations of populations in environment *i*, labelled *j* = 1, 2, …, *nj*.
***α***_*i*_	Vector of Dirichlet distribution parameters for environment *i*, given by (*α*_*i*,1_, *α*_*i*,2_, …, *α*_*i*,*K*_), where *α*_*i*,*k*_ is the parameter associated with class *k*.
***α***	Concatenation of the vectors *α*1, *α*2, …, *αm*.
*x*_*i*, *j*, *k*_	Proportion of individuals in observation *j* of environment *i* that belong to class *k*.
***x***	The set of all *xi*,*j*,*k* observations.
*L*(***α***|***x***)	Likelihood of a parameterisation ***α*** given observations *x*.
*D*	Test statistic used in the likelihood ratio test.
*T*_0_	Test statistic used in goodness-of-fit testing from observed data.
*T*_*j*_	Test statistic used in goodness-of-fit testing with data from simulation *j* where *j* = 1, 2, …, *N* and *N* is the number of simulations used.
$\hat{{\boldsymbol{\alpha }}}$	Parameterisation of ***α*** maximising *L*(*α*|*x*) under the alternative hypothesis. Used in power testing.

### Derivation and considerations of the test statistic

We describe how to model multitype population compositions in terms of a Dirichlet distribution before explaining how the test statistic used to compare compositions is obtained. We also address some important considerations when carrying out the likelihood ratio test, including outlining our method for goodness-of-fit testing, designed to ensure that the Dirichlet distribution provides an appropriate model for the observed community compositions.

### Multitype population structures as Dirichlet distributed random variables

The Dirichlet distribution is a standard distribution in statistics in *K* > 2 dimensions which is described by the parameters *α* = (*α*_1_, *α*_2_, …, *α*_*K*_) with *α*_*k*_ > 0 (*k* = 1, 2, …, *K*). A single, random draw from the Dirichlet distribution will give an observation of the form (*x*_1_, *x*_2_, …, *x*_*K*_) where

1 $$\sum _{k=1}^{K}\,{x}_{k}=1\,{\rm{and}}\,{x}_{k} > 0\,{\rm{for}}\,k=1,2,\ldots ,K.$$

The expected value of *X*_*k*_, the *k*^*th*^ component of a random variable distributed as described above, is given by

$${\mathbb{E}}[{X}_{k}]={\alpha }_{k}/{\alpha }_{0}\,{\rm{where}}\,{\alpha }_{0}=\sum _{k=1}^{K}\,{\alpha }_{k}.$$

Note that the expected values do not change if each of the *α*_*k*_ parametrising the Dirichlet distribution are multiplied by some constant *c* > 0. However, the variance is affected with larger *α*_0_ values resulting in smaller variances. Specifically,

$${\rm{Var}}({X}_{k})=\frac{{\alpha }_{k}({\alpha }_{0}-{\alpha }_{k})}{{\alpha }_{0}^{2}({\alpha }_{0}+1)}.$$

Now suppose that we have an observation of a multitype population containing *K* classes of individuals with *y*_*k*_ individuals in each class (*k* = 1, 2, …, *K*). For *k* = 1, 2, …, *K*, let *x*_*k*_ denote the proportion of individuals of class *k* within the population, given by

$${x}_{k}={y}_{k}/{y}_{0}\,{\rm{where}}\,{y}_{0}=\sum _{k=1}^{K}\,{y}_{k}.$$

Since the *x*_*k*_ meet the conditions set out in (1), we propose that the proportions of individuals in each class can be considered to be a random draw from a Dirichlet distribution with unknown parametrisation ***α***.

### Likelihood Ratio Test

Suppose our multitype population has *K* classes and we have *m* environments of interest. For *i* = 1, 2, …, *m*, let *n*_*i*_ be the number of observations of population compositions in the *i*^*th*^ environment. Let *x*_*i*,*j*,*k*_ denote the proportion of individuals in class *k* in observation *j* from the *i*^*th*^ environment (*i* = 1, 2, …, *m*; *j* = 1, 2, …, *n*_*i*_; *k* = 1, 2, … *K*). We assume that the composition of a randomly selected population in environment *i* follows a Dirichlet distribution with parameters ***α***_*i*_ = (*α*_*i*,1_, *α*_*i*,2_, …, *α*_*i*,*K*_). Let ***α*** be the concatenation of the vectors ***α***_1_, ***α***_2_, …, ***α***_*m*_ and ***x*** denote the set of all observations *x*_*i*,*j*,*k*_. The likelihood of a parametrisation ***α*** given a set of observed population compositions ***x*** is given by

2 $$L({\boldsymbol{\alpha }}|{\boldsymbol{x}})=\prod _{i=1}^{m}\,\prod _{j=1}^{{n}_{i}}\,\frac{1}{B({{\boldsymbol{\alpha }}}_{i})}\,\prod _{k=1}^{K}\,{x}_{i,j,k}^{{\alpha }_{i,k}-1}$$

where *B*(***v***) denotes the multivariate beta function, applied to the vector ***v***.

We wish to test the following nested hypotheses:

$$\begin{array}{ll}{H}_{0}: & {\alpha }_{i}={\alpha }_{j}\,i,j=1,2,\ldots ,m\\ {\rm{vs}}\,{H}_{1}: & {\alpha }_{i}\ne {\alpha }_{j}\,{\rm{for}}\,{\rm{some}}\,i\ne j.\end{array}$$

Let *L*_0_ be the restricted maximum value of *L*(***α***|***x***) under *H*_0_ and *L*_1_ be the unrestricted maximum value of *L*(***α***|***x***) under *H*_1_. Our test statistic is given by

$$D=-\,2\,\mathrm{log}\,(\frac{{L}_{0}}{{L}_{1}}).$$

By standard statistical theory, *H*_0_, *D* follows a ${\chi }_{(m-1)K}^{2}$ distribution, since (*m* − 1) *K* is the number of additional parameters under our alternative hypothesis.

Both the Wald’s and likelihood ratio tests produce test statistics which should follow a ${\chi }_{(m-1)K}^{2}$ under *H*_0_, however this is an *asymptotic* result based on the number of samples from each environment being large. The main reason for the likelihood ratio test being more reliable for smaller sample sizes is that, whilst both tests assume the test statistic takes a chi-squared distribution under *H*_0_, the Wald’s test also relies on assuming that the standard error of parameter estimates is known (see *S* in^19^). This is an unrealstic assumption for smaller sample sizes, hence the value of having likelihood ratio and randomisation tests as alternatives.

Should a randomization procedure be required due to extremely limited replications, as described in the Results section, this can be achieved by calculating *D* as described above for the original data. A further set of test statistics for randomized data can be calculated by randomly permuting the environments associated with the results from each of the samples in the data. The proportion of test statistics from the randomized data that are greater than the value *D* from the original data provide a p-value for the hypothesis test outlined above. Results quoted in this paper are from randomization procedures with 5000 trials.

Code for calculating the test statistic and its associated p-value in R Statistical Software (either by comparison to the relevant *χ*^2^ distribution or by randomization) have been made available on GitHub as described below. Further detail on the derivation and computation of maximum likelihood estimators form a Dirichlet distribution may be found in the note by Minka^28^.

### Goodness-of-fit Testing

The likelihood ratio test outlined above determines which of two models based on the Dirichlet distribution best describes our data. However, our test is based on the assumption that population composition can be described by the Dirichlet distribution, and not require further parameterization, e.g. through a Dirichlet-multinomial distribution. We describe a test to determine whether the selected Dirichlet distribution model provides an adequate fit to the given data.

Standard goodness-of-fit tests such as Pearson’s chi-squared test or the Kolmogorov-Smirnov test are only applicable to one-dimensional distributions. Since the Dirichlet distributions that we are testing are *K*-dimensional (*K* > 2), such tests are inappropriate and thus we use an ad-hoc test best on likelihoods to generate a p-value for goodness-of-fit. The test compares the likelihood given the observed data to likelihoods given simulated data from the proposed distribution with the same parametrisation.

The test is designed as follows. Select a Dirichlet distribution model with parametrisation ***α*** to model the observed data. Calculate the (log-) likelihood of the parametrisation given the observed data using (2) and denote this value by *T*_0_. Choose a suitably large integer *N* and simulate *N* copies of the data, using the same *K*, *m* and *n*_*i*_ (*i* = 1, 2, …*m*) as in the observed dataset. For *j* = 1, 2, …, *N* let *T*_*j*_ be the likelihood of ***α*** given the *j*^*th*^ simulated data set.

For this test, our null hypothesis is that our observed data come from Dirichlet distribution model with parametrisation ***α***. Under this hypothesis, the likelihood of ***α*** given the observed data, *T*_0_, should not be significantly different to the *T*_*j*_ (*j* = 1, 2, …, *N*), the likelihoods of ***α*** given the simulated data. Specifically, the proportion of the *T*_*j*_ < *T*_0_ can be used as a p-value for testing the null hypothesis, since *T*_0_ will be smaller than most of the *T*_*j*_ if the proposed Dirichlet distribution provides a poor fit to the observed data.

### Power Testing

To perform a test estimating the power of future experiments we assume that the alternative hypothesis *H*_1_ is true and specifically, assume that the population compositions are described by $\tilde{{\boldsymbol{\alpha }}}$, where $\tilde{{\boldsymbol{\alpha }}}$ is the value of ***α*** which maximises *L*(***α***|***x***) under *H*_1_ for our original data.

The power of a future experiment may be achieved by selecting a significance value for future tests (usually 5%), a number of replications of the experiment in each environment *n*, and, as with the goodness-of-fit testing, a suitably large integer *N* which denotes the number of simulations to run. Simulate data with *n* replications in each of the *K* environments by simulating *n* copies of each of the *K* Dirichlet distributions described by the parameterisation $\tilde{{\boldsymbol{\alpha }}}$. This creates a single simulation of new data with *n* replications in each environment which can then be put through the likelihood ration test. If the resulting p-value from this test is less than the significance value selected then this is recorded as a success. The proportion of successes in *N* replications of this procedure provides an estimate of the power of a future experiment with *n* replications in each environment.

*Note that the test described here is computationally expensive*. *The attached R code will run much slower if the user includes power testing options*.

### Further Considerations

In our example analysis we make two important decisions which affect both the test statistic and the *χ*^2^ distribution that it is compared to. These are to only consider classes within the multitype population that account for at least 1% of the population in at least one of the samples and to only include classes which are present across all samples.

We are generally interested in differences between the more abundant classes in a given multitype dataset. From the derivation of the likelihood ratio test given earlier in this section, *D*, follows a ${\chi }_{(m-1)K}^{2}$ distribution under *H*_0_. Suppose *K*′ < *K* of the classes fail to meet a minimum percentage threshold such as the 1% given above. The number of degrees-of-freedom associated with the test statistic will reduce by (*m* − 1) (*K*′ − 1) if these classes are aggregated, which will increase the power of the test if there are differences between the more abundant classes between environments. Aggregation of classes may be useful to prevent overfitting if the number of classes is large in comparison to the number of samples. The function to perform the Dirichlet likelihood ratio test given in the code has an option to include a *minimum proportion*. This is a value between 0 and 1 and is the minimum percentage threshold described above, divided by 100. For example, the 1% threshold used here would be described by a minimum proportion of 0.01. The default setting in the code is for the minimum proportion to be 0 and thus not be included.

The use of a 1% threshold in our examples in largely driven by this being a standard the field for analyses of compositional metagenomics data^19^. The choice of threshold affects the dimensionality of the data. A larger threshold will reduce *K* and thus give more reliable results from the use of the *χ*^2^ distribution, however this will also reduce the amount of information available, reducing the power of the test. A minimum proportion may be chosen to allow the *χ*^2^ distribution to be used over the randomization procedure using the guidance given below. The number of classes *K* is also the most important factor in determining the computational expense of the code and so a larger threshold may also be used to combat this issue.

The use of classes that are present across all samples out can be seen by considering the effect on (2) of having some *x*_*i*,*j*,*k*_ = 0. Specifically *L*(***α***|***x***) would be equal to 0 regardless of the choice of ***α***. As such we aggregate all classes that are not ever present. If this aggregated class still contains a zero sample then it is deleted and the data adjusted accordingly so that the compositions in each sample sum to 1. There is an option in the code to remove the need for ever present classes, which replaces all zeros with a small value (approximately 10^−16^) however, results from tests exercising this option should be quoted with extreme caution.

### *χ*^2^ Distribution vs Randomization for generation of p-values

A decision on what constitutes extremely limited replications and thus requires use of randomization testing should be made by comparing the number of samples in the data set, $n={\sum }_{i=1}^{m}\,{n}_{i}$, with the dimensionality of the data, *mK*. Table 2 summarises the results from earlier and provides dimensionality context for each dataset.

**Table 2:** Summary of results for data used in this paper with number of samples, *n*, and dimensionality, *mK*, of each dataset.

Data	*χ*^2^ p-value	Randomization p-value	*n*	*mK*
Soil ARG	1.03 × 10^−125^	0.0002	24	64
Soil Bacteria	1.32 × 10^−19^	0.0266	23	96
Sheep ARG	0.057	0.586	12	27
Sheep Bacteria	0.597	0.898	12	15
Root Bacteria	2.81 × 10^−286^	NA	354	42

If $n\gg mK$, such as for the root bacteria data, the asymptotic theory of the likelihood ratio test holds and, so the use of a *χ*^2^ distribution is preferred to the less powerful and much more computationally expensive randomization procedure. If *n* < *mk* then we see situations such as those for our smaller datasets above where the p-value derived from the *χ*^2^ distribution is much smaller than that from the randomization. Here, a randomization procedure is preferred since the *χ*^2^ approach is too sensitive.

In order to investigate the relationship between sample size and dimensionality further, a series of randomization and likelihood ratio tests were performed on subsets of the root bacteria data. In particular it was found that taking 20 random samples from each site and using a minimum proportion of 0.1 (10%) gave a p-value of 1.09 × 10^−31^ using the *χ*^2^ distribution and 0 under 10000 randomization trials. In this test *n* = 40 and *mK* = 20 and the results suggest that the *χ*^2^ method is not too sensitive here. Note that the 10% threshold used here was purely to reduce dimensionality for illustrative purposes.

### Metagenomics ARG Discovery Methods

This animal study including all methods and protocols was reviewed and approved by the University of Nottingham, School of Veterinary Medicine and Science ethical review committee ERN: 1144 140506 (Non ASPA).

### Isolation of DNA from ovine interdigital swabs

Samples were collected from four sheep kept on the University of Nottingham, Sutton Bonington Campus farm during routine husbandry checks during the winter (November), spring (May) and summer (August) 2015/16. Sterile nylon flock swabs were used for the collection of samples from the interdigital space of sheep and stored in liquid amies solution at 5 °C overnight. The swabs were processed following the methodology of^29^.

### DNA isolation and sequencing

DNA was isolated using the Qiagen Cador Pathogen Mini Kit, following the manufacturers guidelines and eluted in 60 *μ*l of elution buffer. The DNA samples were quantified using the Qubit 3.0 and dsDNA high sensitivity dye. Quantified DNA was sent to Leeds Genomics (Leeds Univerity, U.K.) and prepared for sequencing using the NEB Library preparation kit and sequenced on the Illumina HiSeq 3000 at an approximate read depth of 50 million reads per sample.

### Analysis of sequence data

Raw reads were analysed for sequence adaptors using trimmomatic^30^ and clipped if necessary, the reads were then error corrected using the SGA k-mer based approach^31^. The corrected reads were firstly analysed using MetaPhlAn 2^3^ to identify bacteria present in the samples and then parsed against the MegaRes database^32^ using Diamond^33^ to identify any antimicrobial resistance genes present. Finally BacMet^34^ was used to identify any resistance genes associated to non-antibiotic elements.

## Data Availability

Raw sequences from the sheep hoof experiment have been deposited at the NCBI BioProject with accession number PRJNA500434. Derived data on bacterial taxonomic groups and antimicrobial resistance gene abundances have been deposited in GitHub at https://github.com/LMShaw/DirtyGenes. R code for the DirtyGenes analysis, including a user guide, has also been deposited in Github at https://github.com/LMShaw/DirtyGenes.
